# Characterization of Yeast Isolated from the Gut Microbiota of Tunisian Children with Autism Spectrum Disorder

**DOI:** 10.3390/jof10110730

**Published:** 2024-10-22

**Authors:** Mariem Chamtouri, Abderrahmen Merghni, Katherine Miranda-Cadena, Nabil Sakly, Naoufel Gaddour, Clara G. de Los Reyes-Gavilán, Maha Mastouri, Elena Eraso, Guillermo Quindós

**Affiliations:** 1 Department of Microbiology and Biochemistry of Dairy Products, Instituto de Productos Lácteos de Asturias (IPLA-CSIC), 33300 Villaviciosa, Spain; myriam.chantouri88@gmail.com (M.C.); greyes_gavilan@ipla.csic.es (C.G.d.L.R.-G.); 2 Laboratory of Transmissible Diseases and Biologically Active Substances LR99ES27, Faculty of Pharmacy, University of Monastir, Monastir 5000, Tunisia; mastourimaha@yahoo.fr; 3 Laboratory of Antimicrobial Resistance LR99ES09, Faculty of Medicine of Tunis, University of Tunis El Manar, Tunis 1068, Tunisia; abderrahmen_merghni@yahoo.fr; 4 Department of Immunology, Microbiology and Parasitology, Faculty of Medicine and Nursing, University of the Basque Country, UPV/EHU, 48080 Bilbao, Spain; katherine.miranda@ehu.eus (K.M.-C.); guillermo.quindos@ehu.eus (G.Q.); 5 Laboratory of Medical and Molecular Parasitology-Mycology (code LR12ES08), Department of Clinical Biology B, Faculty of Pharmacy, University of Monastir, Monastir 5000, Tunisia; saklynabil@yahoo.fr; 6 Unit of Child Psychiatry, Monastir University Hospital, Monastir 5000, Tunisia; naoufel.gaddour@gmail.com; 7 Diet, Microbiota and Health Group, Instituto de Investigación Sanitaria del Principado de Asturias (ISPA), 33011 Oviedo, Spain

**Keywords:** autism, *Candida*, gut microbiota, antifungal susceptibility, virulence

## Abstract

Research on the microbiota–gut–brain axis in autism has primarily focused on bacteria, with limited attention to fungi. There is a growing interest in understanding the involvement of fungi, particularly *Candida*, in patients with autism spectrum disorder. The aim of this study was to assess the prevalence, antifungal susceptibility profiles and virulence factors of *Candida* isolates from the guts of Tunisian children with autism. Twenty-eight children with autism and forty-six controls were enrolled. *Candida* isolates from the faecal samples were identified using biochemical and molecular methods; antifungal susceptibility testing was determined by the EUCAST broth microdilution method and virulence factors, including biofilm formation, cell surface hydrophobicity and phospholipase and proteinase activities, were assessed in vitro. As a result, *Candida* was detected in 13 children with autism (46.4%) and 14 control children (30.4%). *Candida albicans* was found to be the most common species isolate in the faeces of both groups of children. Antifungal susceptibility profiles showed that one *Candida* isolate was resistant to amphotericin B and anidulafungin (3.7%), six were resistant to micafungin (22.2%) and five were resistant to fluconazole (18.5%). All *Candida* isolates were biofilm producers. Of the twenty-seven isolates, only four showed phospholipase activity (14.8%), eight showed aspartyl-proteinase activity (29.6%) and nine were hydrophobic (33.3%). These results highlight the presence of *Candida* in the guts of children with autism, as well as the ability to express multiple virulence factors and the antifungal resistance, and they emphasize the need for further studies to confirm intestinal *Candida* colonization and its potential role in autism.

## 1. Introduction

Autism spectrum disorder (ASD) is a neurodevelopmental disorder characterized by impaired social interaction and communication, as well as restrictive and repetitive behaviours or interests. ASD is one of the most common childhood mental disorders, with varying degrees of severity. The most recent global prevalence of autism is estimated at 0.76%, which accounts for around 16% of the global child population [1,2,3]. The etiopathogenesis of autism remains poorly understood, as this disorder involves genetic abnormalities, dysregulation of the immune system, inflammation, environmental factors and modifications of gut microbiota. Gastrointestinal (GI) symptoms, such as constipation, abdominal pain, flatulence and diarrhoea are quite common in patients with ASD and are strongly associated with the severity of ASD [4,5,6]. The frequently reported GI problems may be associated with an altered gut microbiota, highlighting a close connection between gut and brain, the so-called “microbiota–gut–brain axis”, a physiological bidirectional network of communication between the gut and the brain [7,8]. Indeed, the gut microbiota could influence the central nervous system through multiple mechanisms, including neural, immune and endocrine ones, and through the production of microbial toxins [4]. Consistent evidence of microbiota dysbiosis in ASD has been shown in recent years. Despite the heterogeneity of results among studies, most of them observed an increase in the presence of genera such as *Clostridium*, *Sutterella* and *Faecalibacterium* and a lower proportion of *Bifidobacterium* [9,10,11]. Furthermore, although several studies have assessed the gut microbiota profile in ASD children, to our knowledge, few studies have analysed the gut mycobiota and its potential role in ASD [4,12]. Some reports showed an increased abundance of *Candida* species in the faecal samples of children with ASD [13,14,15]. *Candida* colonization can cause malabsorption of carbohydrates and minerals, and can release ammonia and toxins, which have been reported to be associated with some autistic behaviour [16,17]. These findings suggest that *Candida* may be involved in the ASD pathogenesis. Therefore, an extensive understanding of the role of intestinal *Candida* colonization in ASD development is needed.

The present study aimed to determine the prevalence of *Candida* colonization in the guts of Tunisian children with ASD. In addition, the distribution of *Candida* species, in vitro antifungal susceptibility of the collected isolates and the production of several virulence factors, such as biofilm formation, cell surface hydrophobicity and proteinase and phospholipase activities, were evaluated. We also compared the clinical data and investigated the association between ASD severity, GI disorders and *Candida* intestinal colonization among children with ASD and control (CC) groups.

## 2. Subjects and Methods

### 2.1. Subjects

A total of 28 children with ASD aged between 4 and 10 years old were recruited at the Unit of Child and Adolescent Psychiatry, Department of Psychiatry, Fattouma Bourguiba University Hospital, Monastir, Tunisia between 2019 and 2020. During the same period, 46 age-matched children were enrolled from siblings and children of the general population as a control group. ASD patients were diagnosed according to the diagnostic and statistical manual of Mental Disorders (DSM-5) criteria [1], the Autism Diagnostic Inventory-Revised (ADI-R) [18] and the Autism Diagnostic Observation Schedule-2 (ADOS-2) [19]. The severity of autism was assessed by the childhood autism rating scale (CARS) [20]. Exclusion criteria included associated pathologies that can lead to risks: neurological disorders not strictly associated with autism, type 1 diabetes, genetic syndromes, coeliac disease, food intolerance or inflammatory bowel disease. Subjects in this study were not treated with antibiotics, antifungal drugs, probiotics and/or prebiotics for at least one month before sampling. The clinical data of children with ASD and control children were collected in Table 1.

### 2.2. Clinical Diagnosis

The DSM-5 is a reference guide for mental health professionals to diagnose, classify and identify mental health disorders. It was published in May 2013 by the American Psychiatric Association (APA) [1]. The ADI-R is a structured interview administered to parents and/or caregivers of children with suspected ASD. It contains 93 items relating to social interaction, communication and restricted, repetitive and stereotyped behaviours. This exam usually takes about 2 or 3 h to complete [18]. The ADOS is a standardized protocol for diagnosing ASD. It consists of a series of activities that evaluate communication, reciprocal social interaction, imagination and creativity. The ADOS exam lasts approximately 40 min [19]. The CARS is a 15-item behavioural rating scale used for assessing the severity of ASD. The CARS score can range between 15 and 60. Children who have a score of less than 30 are in the non-autistic range. Mild to moderate autism is indicated by a score of 30 to 36, whereas severe autism is indicated by a score of 37 to 60 [20].

### 2.3. Faecal Sample Collection and Yeast Isolation

A total of 74 faecal samples were collected from children in sterile containers by the first investigator and transported to the laboratory for processing on the same day. From each sample, 1 g of faeces was resuspended in 10 mL of 0.9% sterile saline solution and homogenized by vortexing. Ten µL of this mixture was inoculated on Sabouraud dextrose agar medium with chloramphenicol and incubated aerobically at 37 °C for 24–48 h.

### 2.4. Candida Identification

*Candida* identification was performed by morphologic, biochemical and molecular methods [21]. Isolated colonies with various morphologies were Gram stained and observed under the microscope. Each colony type was subcultured for purity and stored at −20 °C for further analysis. Thawed isolates were cultured for 48 h at 37 °C on Candida chromogenic (Condalab, Madrid, Spain) and ChromID Candida (bioMérieux, Craponne, France) agars and identification was carried out considering colony colour and morphology. Colonies that appeared with colours other than green and blue on the selective media used were respectively identified using API ID32C (bioMérieux).

All the isolates that grew as green colonies on Candida chromogenic agar and blue colonies on ChromID Candida were tested to distinguish between *Candida albicans*, *Candida dubliniensis* and *Candida africana* by amplification of the hyphal wall protein 1 gene (HWP1) based on the different amplicon sizes—700 bp for *C. africana*, 569 bp for *C. dubliniensis* and approximately 941 bp for *C. albicans* (Figure S1) [21]. Briefly, the isolates were plated on Sabouraud dextrose agar (Difco, Becton Dickinson, Franklin Lakes, NJ, USA) and incubated overnight at 37 °C. Then, a single colony was cultured in yeast extract peptone dextrose broth (YEPD, Panreac, Barcelona, Spain) and incubated for 48 h at 30 °C in shaking conditions. DNA was extracted from *Candida* isolates using a DNeasy Ultraclean Microbial Kit (QIAGEN, Germantown, MD, USA) following the manufacturer’s instructions. A Nano Drop ND-1000 spectrophotometer (Thermo Fisher Scientific, Waltham, MA, USA) was used to measure DNA concentration and purity. Extracted DNA was stored at −20 °C until use. The HWP1 gene was amplified using the primers CRR-f: 5′-GTTTTTGCAACTTCTCTTTGTA-3′ and CRR-r: 5′-ACAGTTGTATCATG TTCAGT-3′ and the PCR was performed according to the protocol described by Romeo et al. [21]. PCR reaction conditions were as follows: denaturation at 95 °C for 5 min, 30 cycles of denaturation at 94 °C for 45 s, primer annealing at 58 °C for 40 s and extension at 72 °C for 55 s, followed by a final extension at 72 °C for 10 min in a C 1000TM Thermal Cycler (Bio-Rad, Hercules, CA, USA). The PCR products were separated by electrophoresis on a 1.5% agarose gel stained with Gel Red (Biotium, Fremont, CA, USA) and then visualized on a UV transilluminator. A DNA ladder (HyperladderTM 50 bp [50–2000 bp]; Bioline, London, UK) was used as a molecular weight standard. DNA of *C. albicans* NCPF 3153, *C. dubliniensis* NCPF 3949 and *C. africana* ATCC MYA-2669 were included as positive controls.

### 2.5. In Vitro Antifungal Susceptibility Testing

The activity of six antifungal drugs against all *Candida* isolates were tested: amphotericin B (Sigma-Aldrich, Madrid, Spain), micafungin (Astellas Pharma Inc., Tokyo, Japan), anidulafungin (Pfizer SA, Madrid, Spain), fluconazole (Pfizer SA), isavuconazole (Basilea Pharmaceutica International, Allschwil, Switzerland) and ibrexafungerp (Scynexis Inc., Jersey City, NJ, USA). Stock solutions of each drug were prepared in dimethyl sulfoxide (DMSO) and stored at −80 °C until use. The final drug concentration ranged from 0.008 to 4 mg/L for amphotericin B, anidulafungin, micafungin and isavuconazole, from 0.125 to 64 mg/L for fluconazole and from 0.016 to 16 mg/L for ibrexafungerp. The minimal inhibitory concentrations (MICs) of the antifungal drugs were determined by broth microdilution method in 96-well flat-bottom microtiter plates, in RPMI 1640 medium, according to EUCAST guidelines [22]. Prior to the experiment, an inoculum was prepared in sterile distilled water for each isolate obtained from an overnight culture at 37 °C. The final inoculum, between 0.5–2.5 × 10^5^ CFU/mL, was added to the microtiter plates. The plates were then incubated at 37 °C for 24 h and the absorbance was measured at a wavelength of 450 nm using an Infinite F50 spectrophotometer (Tecan, Männedorf, Switzerland). Two reference strains *Candida krusei* ATCC 6258 (currently *Pichia kudriavzevii*) and *Candida parapsilosis* ATCC 22019 were used as quality controls. MICs were read at 24 h and defined as the antifungal concentration that inhibited at least 50% of *Candida* growth, except for amphotericin B, for which the MIC evaluated 90% of growth inhibition. For each species and antifungal, descriptive statistics, including MIC ranges and geometric mean MICs (GM), were calculated. The interpretation of susceptibility was performed according the EUCAST clinical breakpoints [23]. No interpretive breakpoints are available for isavuconazole and ibrexafungerp.

### 2.6. Biofilm Development

Prior to the experiment, *Candida* strains were inoculated in YEPD broth and were incubated overnight under orbital shaking 120 rpm at 30 °C. Cells were harvested and washed three times with sterile phosphate buffered saline solution (PBS, Sigma-Aldric). After cell counting by microscopy using a Burker haemocytometer, cell suspensions of each *Candida* isolate were adjusted to a final concentration of 10^6^ cells/mL, with RPMI 1640 medium supplemented with L-glutamine and buffered with morpholine propane-sulfonic acid (MOPS, Sigma-Aldrich) to pH 7. *Candida* biofilm formation was performed on sterile, flat-bottomed honeycomb 100-well microtiter plates (Labsystems, Vantaa, Finland). One hundred µL of each adjusted cell suspension was transferred into the wells of the plate. The microtiter plates were then incubated at 37 °C. After 24 h and 48 h, the wells were washed with 100 µL of sterile PBS to remove unattached and weakly attached cells [24].

Biofilm biomass was quantified using crystal violet (CV) staining [25]. After washing, the biofilms were dried at room temperature for 30 min, then a volume of 100 µL of 0.4% CV solution (Merck, Darmstadt, Germany) was added to each well and stained for 20 min. Afterwards, the excess CV was removed by rinsing the plates twice with 250 µL of sterile distilled water. Finally, the bound CV was released by adding 150 µL of 33% acetic acid. Biofilm metabolic activity was measured using a colorimetric method based on the reduction of 2,3-bis-(2-methoxy-4-nitro-5-sulfophenyl)-5-[(phenylamino)-carbonyl]-2H-tetrazolium hydroxide (XTT, Sigma-Aldrich) [26]. Briefly, XTT was prepared as a saturated solution at a concentration of 0.5 g/L in Ringer’s lactate. The solution was filter-sterilized using a 0.22 µm-pore-size filter (Sarstedt, Nümbrecht, Germany) and aliquots were stored at −70 °C until use. Before each assay, 100 μL of an aliquot of XTT mixed with 1 μM menadione was added to each well and the microplates were incubated in the dark at 37 °C for 2 h.

Absorbances were measured at two time points, 24 and 48 h, using a BioScreen C MBR microplate reader (Growth Curves Ltd., Turku, Finland) at a wavelength of 600 nm for biofilm biomass and 492 nm for biofilm metabolic activity. Two independent experiments were performed with five replicates for each condition, and the reference strains *C. albicans* SC5314 and *C. albicans* Ca2 hypha-defective mutant (graciously donated by Professor Antonio Cassone, Instituto Superiore di Sanità, Rome, Italy) were included as positive and negative controls, respectively.

### 2.7. Cell Surface Hydrophobicity Assay

Cell surface hydrophobicity (CSH) was determined using the microbial adhesion to hydrocarbon (MATH) test [27,28]. Briefly, the yeast cells grown overnight at 30 °C in YEPD broth were washed twice with sterile PBS and resuspended in the same buffer to adjust an absorbance between 0.4 and 0.5 at 600 nm (A0). Three mL of this yeast cell suspension was overlaid with 0.4 mL of n-hexadecane (Sigma-Aldrich). After vigorous vortexing, aqueous and organic phases were allowed to separate for 10 min at 30 °C and the absorbance of the aqueous phase was measured at 600 nm (A1). The percentage of hydrophobicity was calculated according to the following equation: percentage of CSH = [1 − (A1/A0)] × 100. The highly hydrophobic strains exhibited CSH values of more than 50%, and the moderately hydrophobic strains had CSH values ranging between 20 and 50%. Hydrophilic strains had CSH values of less than 20% [29].

### 2.8. Determination of Phospholipase and Proteinase Activity

Isolates were grown on Sabouraud dextrose agar plates overnight at 37 °C and cells were then suspended in sterile saline solution to a final suspension of 10^7^ cells/mL. Ten µL of this suspension was inoculated on each specific medium.

Phospholipase activity was evaluated following the method described by Polak [30], using malt agar plates supplemented with 1 M NaCl, 5 mM CaCl_2_ and 8% sterile egg yolk emulsion [31]. The strain *C. albicans* UPV/EHU 04-125 was included as positive control. Aspartyl proteinase activity was assessed using bovine serum albumin (BSA) agar plates [32]. Briefly, the BSA medium consisted of 1.17% yeast carbon base (Difco), 0.01% yeast extract (Condalab) and 0.2% BSA (Sigma-Aldrich). The medium was sterilized by filtration and mixed with a stock solution of autoclaved 2% bacto-agar (Difco). *C. dubliniensis* UPV/EHU 00-134 was used as positive control [33]. After inoculation, the plates were incubated at 37 °C for 6 days. The phospholipase activity (Pz) value was determined as the ratio of the diameter of the colony to the total diameter of the colony plus precipitation zone and was classified as follows: 0.35–0.5 (high producers); 0.51–0.74 (moderate producers); 0.75–0.9 (low producers) and 1 (non-producers). The aspartyl-proteinase activity was established as the diameter of a transparent halo around growing colonies. *Candida* isolates were classified as non-producers (when no visible halo was present), moderate producers (when the diameter of the halo was 1–2 mm) and high producers (when the diameter of the halo was >2 mm) [33]. Each isolate was assayed in triplicate.

### 2.9. Statistical Analysis

Statistical analysis of quantitative and qualitative data, including descriptive statistics, was performed. Frequencies comparison of categorical data was performed with Chi-square test or Fisher’s exact test. The intergroup differences of continuous data were conducted by Student’s test when data showed a normal distribution and Mann–Whitney nonparametric test when data did not show a normal distribution. All tests were two sided and a *p*-value < 0.05 was considered as statistically significant. Data were analysed using SPSS software (version 26) and figures were constructed using GraphPad Prism (version 8.0.2).

## 3. Results

### 3.1. Clinical Characteristics of the Study Population

As shown in Table 1, there were no statistically significant differences in age and gender between the ASD and CC groups. Moreover, GI disorders did not significantly differ when comparing both groups, except for constipation: constipation was more frequent in the ASD group than the CC group (60.7% vs. 17.4%, respectively, *p* = 0.000).

### 3.2. Association between ASD Severity and Clinical Data in the ASD Group

**Figure 1:**
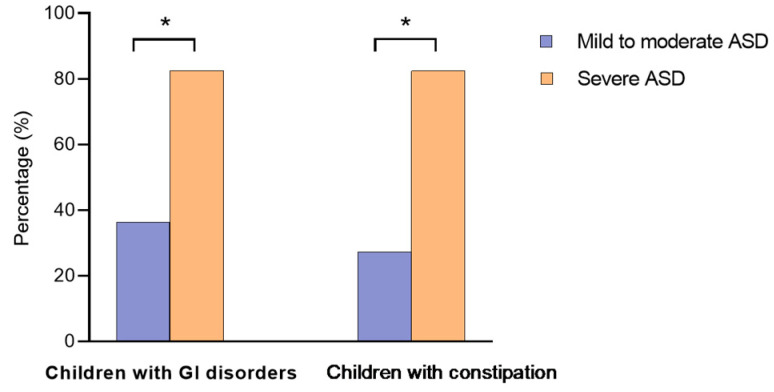
Presence of GI disorders and constipation in children suffering from mild to moderate ASD and severe ASD. GI, gastrointestinal; ASD, autism spectrum disorder. Asterisks (*) indicate significant differences between the two ASD subgroups: mild to moderate and severe.

According to the CARS score, children with ASD were divided into two groups: children with mild–moderate ASD (*n* = 11) and children with severe ASD (*n* = 17). We found significant associations between GI disorders and ASD severity (*p* = 0.020): a higher percentage of children suffering from severe ASD (82.4%) had GI disorders. In contrast, only 36.4% of children with mild to moderate autism presented GI disorders (Figure 1). Constipation was also associated with ASD severity (*p* = 0.006). Children with severe ASD were more likely to present constipation than children with mild to moderate ASD (82.4% vs. 27.3%) (Figure 1). No significant differences were observed between ASD severity and other clinical parameters.

### 3.3. Prevalence of Candida in the Gut Microbiota of Children with ASD and Control Children

**Figure 2:**
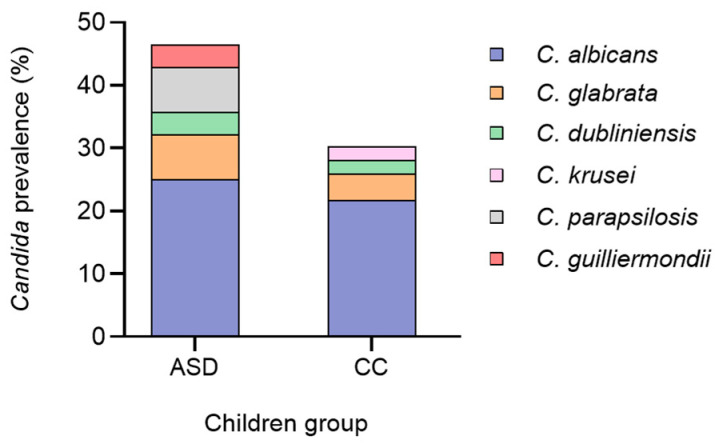
Prevalence of *Candida* in stools of children with ASD and CC group. ASD, autism spectrum disorder; CC, control children. *C. glabrata* (*n* = 2), *C. parapsilosis* (*n* = 2), *C. dubliniensis* (*n* = 1) and *C. guilliermondii* (*n* = 1) were non-*C. albicans* species isolated in stools of children with ASD. *C. glabrata* (*n* = 2), *C. dubliniensis* (*n* = 1) and *C. krusei* (*n* = 1) were non-*C. albicans* species isolated in stools of the control children group.

*Candida* was detected in 13 children with ASD (13 out of 28, 46.4%) and in 14 control children (14 out of 46, 30.4%) (Figure 2, Table S1). The difference in *Candida* prevalence between the two groups of children was not statistically significant (*p* = 0.166). The species *C. albicans* was the most frequent in faecal samples of both ASD children (*n* = 7 out of 13 isolates; 53.8%) and the CC group (*n* = 10 out of 14 isolates; 71.4%). The most common non-*C. albicans* species detected in the stools of the group with ASD were *Candida glabrata* (currently *Nakaseomyces glabratus*; *n* = 2; 15.4%), *C. parapsilosis* (*n* = 2; 15.4%), *C. dubliniensis* (*n* = 1; 7.7%) and *Candida guilliermondii* (currently *Meyerozyma guilliermondii*; *n* = 1; 7.7%). As for the control group, the identification of non-*C. albicans* species yielded detection of *C. glabrata* (*n* = 2; 14.3%), *C. dubliniensis* (*n* = 1; 7.1%) and *C. krusei* (*n* = 1; 7.1%). There were no significant differences in the distribution of *Candida* species or in the diversity of these species between children with ASD and controls (*p* = 0.480). No cultures with more than one species were detected.

### 3.4. Association Between Candida Gut Colonization and the Clinical Data of the ASD Group

In the group of children with ASD, there were no statistically significant differences between faecal specimens with positive or negative *Candida* cultures regarding their gender or age. Similarly, no statistically significant differences in *Candida* presence were observed between ASD children with or without GI disorders and with or without severe ASD (Table S2).

### 3.5. Antifungal Susceptibility Profile of Candida Isolates

The activities of the six antifungal agents tested against the *Candida* isolates are presented in Table 2. In the group of children suffering from ASD, all *C. albicans* (*n* = 7) were susceptible to amphotericin B, one isolate was resistant to anidulafungin, two were resistant to micafungin and four were resistant to fluconazole. *C. glabrata* isolates (*n* = 2) were resistant to micafungin, susceptible—dose dependent—to fluconazole and susceptible to amphotericin B and anidulafungin. The two isolates of *C. parapsilosis* were susceptible to anidulafungin, micafungin and fluconazole and one isolate was found to be amphotericin B resistant. The isolate of *C. dubliniensis* was susceptible to amphotericin B and fluconazole.

In the CC group, all *C. albicans* isolates (*n* = 10) were susceptible to amphotericin B, anidulafungin, micafungin and fluconazole. Two *C. glabrata* isolates were susceptible to amphotericin B and anidulafungin and resistant to micafungin; one isolate was fluconazole resistant, and one was susceptible, dose dependent. The *C. dubliniensis* isolate was susceptible to amphotericin B and fluconazole and the *C. krusei* isolate was susceptible to amphotericin B and anidulafungin.

Furthermore, low MIC values were found for isavuconazole and they ranged from 0.008 to 0.03 mg/L for *C. albicans* and from 0.008 to 1 mg/L for non-*C. albicans*. MICs of ibrexafungerp against *Candida* isolates were also tested in vitro, ranging from 0.016 to 1 mg/L. The lowest ibrexafungerp MICs were observed against *C. albicans* isolates (GM 0.02 mg/L, MIC range 0.016–0.03 mg/L) and the highest were obtained for the *C. guilliermondii* isolate (MIC = 1 mg/L). Adopting wild-type upper limits recently proposed by Quindós et al. [34] for *C. albicans* (0.5 mg/L), *C. glabrata* (1 mg/L), *C. parapsilosis* (2 mg/L), and for *C. krusei* (4 mg/L), no non-wild-type phenotype for ibrexafungerp was observed, and all isolates were considered susceptible to this antifungal drug.

### 3.6. Biofilm Production by Candida Isolates

Based on optical density values, *Candida* isolates were categorized as high biomass or with high metabolic activity biofilm when the mean absorbance was higher than 0.5, moderate biomass or with moderate metabolic activity biofilm when absorbance ranged from 0.3 to 0.499 and low biomass or with low metabolic activity biofilm when the mean values of absorbance were less than 0.3 for CV and XTT assay [24]. The absorbance was measured on the microtiter plate reader BioScreen C MBR (Growth Curves Ltd., Turku, Finland) at 492 nm and 600 nm to determine metabolic activity and biomass, respectively (Table 3, Table S3). *C. albicans* isolates produced significantly greater amounts of biofilm in vitro than non-*C. albicans* isolates (*p* = 0.001). Furthermore, no statistical differences in biofilm production (*p* = 0.896) and biofilm metabolic activities (*p* = 0.536) were observed in *Candida* isolates between the group with ASD and the CC group.

### 3.7. Cell Surface Hydrophobicity (CSH)

**Figure 3:**
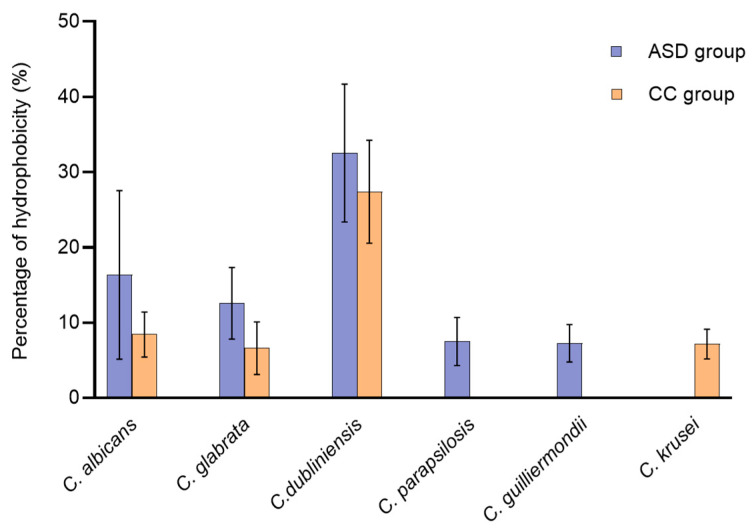
Percentage of *Candida* surface hydrophobicity in the isolates of ASD and CC groups. ASD, autism spectrum disorder; CC, control children.

Hydrophobicity of *Candida* cells was measured for all clinical isolates. However, high intraspecific variability was shown (Figure 3, Table S4). In the group of children suffering from ASD, eight out of thirteen isolates (61.5%) were moderately hydrophobic, whereas in the control group, only one out of fourteen isolates (7.1%) was moderately hydrophobic. When we compared the percentage of CSH mean values of *Candida* isolates between children with ASD and controls, we found that isolates from the group with ASD displayed a higher percentage of hydrophobicity than isolates recovered from the CC group without reaching statistically significant difference (*p* = 0.052).

### 3.8. Phospholipase and Proteinase Activities

The enzymatic activities of *Candida* isolates are shown in Table 4. Of the 27 *Candida* isolates, only four *C. albicans* (14.8%) showed low phospholipase activity. *Candida* strains isolated from children with ASD and the CC group showed similar Pz mean values with no statistically significant differences between them (*p* = 0.937). In addition, eight isolates (29.6%) exhibited moderate aspartyl proteinase activity, of which six were *C. albicans* and two *C. dubliniensis*. No significant difference was found in proteinase production by *Candida* species isolated from the group with ASD and the CC group (*p* = 0.652).

## 4. Discussion

The human gut harbours archaea, bacteria, fungi and viruses—a microbial ecosystem or microbiota that plays a key role in human physiology and health [35,36]. Subsequently, imbalance of the gut microbiota or dysbiosis may contribute to the pathogenesis of several human diseases. GI disturbances observed in children with ASD have been associated with imbalanced composition of the gut microbiota [4]. In addition, the gut microbiota can influence the brain via the microbiota–gut–brain axis. Thus, the intestinal microbiota has recently become one of the major topics of ASD research interest. Although most gut microbiota studies have been mainly focused on bacteria, few studies have evaluated the impact of mycobiota on ASD [4,37]. The latest studies have reported an overgrowth of *Candida* in the gut microbiota of children with ASD [13,14,15,38]. However, little is known about the role of intestinal *Candida* colonization in these patients. Thus, in our study, we used a culture-based method to investigate the prevalence and characteristics of *Candida* species isolated from the gut microbiota of 28 Tunisian children with ASD in comparison with 46 controls.

Despite the widely different rates of GI disorders among studies, constipation was the most prevalent GI disturbance in children with ASD [39,40]. In the current study, constipation was reported in 60.7% of children suffering from autism, compared with 45.7% in healthy children (*p* < 0.05), with a strong association between constipation and the severity of ASD. This finding is consistent with that of other studies [39,41] but is not a generalised observation, and some reports did not find any relationship between GI disorders and autism severity [40,42,43]. This disagreement might be explained by the different methodology used to measure ASD severity: CARS score, Autism Treatment Evaluation Checklist, ATEC, etc. [20,44].

In the current study, there were no significant differences in the prevalence of *Candida* in the faeces of children with ASD in comparison with control children, as reported by Adams et al. [41] and Zou et al. [37]. Conversely, other studies reported a significantly higher prevalence of *Candida* in children with ASD [13,14,38,39]. Nirmalkar et al. suggest that *Saccharomyces cerevisiae* deficiency and *C. albicans* overgrowth are associated with a greater severity of autism in children [45]. Intestinal colonisation by yeasts, mainly of the genus *Candida* or related species, can be influenced by a number of factors, including age, diet, lifestyle and geography [46]. For example, diets high in sugar, excessive alcohol consumption and overuse of antibiotics have been linked to increased *Candida* colonisation. Stress, lack of sleep and smoking can also contribute to the overgrowth of these microorganisms. Finally, geography may play a role in yeast colonisation, as certain species are more common in some regions of the world than in others. Therefore, the detection of *Candida* in faeces by the culture method may lack specificity and/or sensitivity and can lead to false negative results. Even though GI disorders were found in 64.3% of children with ASD, this higher rate did not correlate with *Candida* presence. Moreover, ASD severity was not affected by *Candida* gut colonization. These findings are in line with the study conducted by Iovene et al. [13].

*C. albicans* was more frequent in control children than in children with ASD without a statistically significant difference, which was in good agreement with previous studies [14,37]. In contrast to previous findings [14], we did not isolate *C. tropicalis* and *C. krusei* from children with ASD. However, we found in faecal samples from ASD children other non-*C. albicans* species, such as *C. glabrata*, *C. parapsilosis*, *C. dubliniensis* and *C. guilliermondii*. Differences between studies may be associated with the identification methods used or with dietary and geographical factors. *C. albicans* is a commensal of the human microbiota, but under certain circumstances, including the use of broad-spectrum antibiotics, abdominal surgery or a compromised immune status, causes local and systemic infections [47,48]. The exact role of *Candida* in the GI tract of children with ASD and its potential pathogenicity are not clearly defined.

The present work is the first study to characterize the antifungal susceptibility profile and virulence factors of *Candida* species isolated from the gut microbiota of Tunisian children with ASD. Indeed, only Kantarcioglu et al. [14] and Ahmed et al. [38] investigated the antifungal susceptibility profile of *Candida* isolated from the gut microbiota of children with ASD, but without characterizing its virulence factors. The increasing prevalence of mycoses and the overuse of antifungal drugs have led to the emergence of high rates of resistance among *Candida* isolates worldwide, especially in children [49,50]. Concerning the six common antifungal drugs tested in this study, all *Candida* isolates were susceptible to amphotericin B, but an isolate of *C. parapsilosis* in the group with ASD showed a MIC of 4 mg/L. In fact, resistance to amphotericin B is extremely rare and it is caused by decrease or lack of ergosterol in the fungal cell membrane [51]. Concerning the resistance to echinocandins, only one *C. albicans* isolate was resistant to anidulafungin. However, six isolates (four *C. glabrata* and two *C. albicans*) were resistant to micafungin. This echinocandin resistance observed in *C. albicans* and *C. glabrata* isolates has been previously described [52,53]. Fluconazole is one of the most commonly prescribed antifungal drugs for the treatment of candidiasis. This overuse has led to the emergence of fluconazole-resistant isolates [54]. In the present study, four *C. albicans* isolates from the group with ASD and one *C. glabrata* isolate from the CC group were resistant to fluconazole. Focusing on fluconazole activity in the group with ASD, two previous studies have shown that all *C. albicans* faecal isolates from children with ASD were susceptible to fluconazole, while some non-*C. albicans* isolates showed different antifungal susceptibility patterns [14,38]. These findings indicate that fluconazole should not be used as empiric treatment for candidiasis in children with ASD. Moreover, another azole, isavuconazole, was tested and, although EUCAST does not propose clinical breakpoints for *Candida* species, the observed MICs coincided with those described for susceptible and/or wild-type isolates [23].

The increasing resistance to azoles and echinocandins, in addition to the toxicity of amphotericin B and the lack of the oral bioavailability of polyenes and echinocandins, requires new antifungal agents. Ibrexafungerp is a novel oral and intravenous antifungal agent that inhibits (1-3)-β-D-glucan synthase, a major component of the fungal cell wall [55,56,57]. In the current study, ibrexafungerp showed potent in vitro activity against all *C. albicans* isolates (MIC range 0.016–0.03 mg/L), as previously reported against most species of *Candida* [34,58,59].

By developing multiple virulence factors, *Candida* can play a crucial role in its pathogenicity, adhesion and evasion of host immune responses [60]. In this study, *C. albicans* produced significantly greater amounts of biofilm compared to the other species of *Candida*, as previously described [61,62]. Additionally, biofilm production by the non-*C. albicans* species observed in our study agrees with previous studies with isolates of *Candida* from several body sites [24,28,63]. In fact, biofilms represent a physical barrier for the host immune response and antifungal drugs and can act as a reservoir for future infections [64].

**Figure 4:**
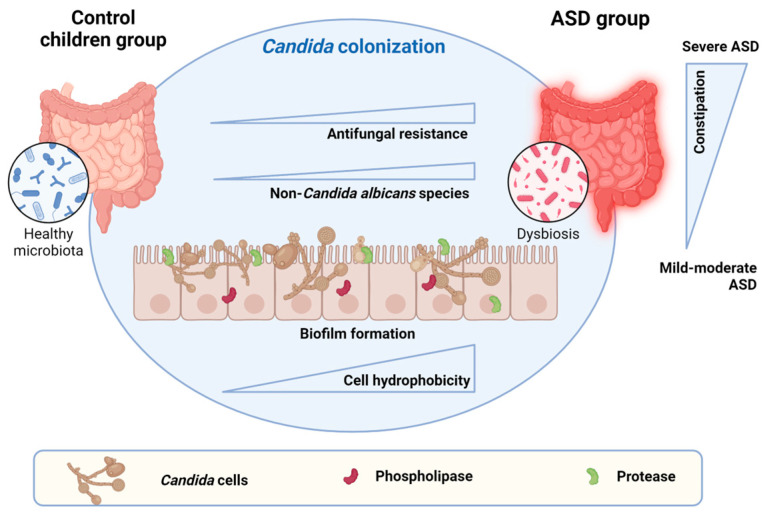
Schematic representation of main findings. *Candida* colonization may play a role in the imbalance in the gut microbiota. *Candida* virulence factors such as cellular hydrophobicity occurred to a greater extent in the ASD group. No significant differences in biofilm production, proteases, phospholipases and species frequency were observed between the groups. However, the presence of antifungal-resistant isolates and non-*C. albicans* species with reduced susceptibility may contribute to dysbiosis and reduce the effectiveness rate of conventional treatments. In addition, the severity of ASD was associated with a higher frequency of constipation (created in https://www.biorender.com/ (accessed on 17 October 2024)).

Cell surface hydrophobicity is considered a virulence factor that plays an important role in *Candida* adherence to host tissues and biomaterials, and in resistance to host immune responses [65]. Our findings demonstrate that most of the species of *Candida* from both groups of children were hydrophilic and only nine *Candida* isolates were moderately hydrophobic (Figure 4).

Another important virulence factor of *Candida* is the secretion of phospholipase and aspartyl-proteinase. These enzymes facilitate adhesion and tissue penetration [66]. Phospholipases facilitate cellular invasion by their ability to hydrolyse one or more ester linkages in glycerophospholipids, which are the major constituents of the host cell envelope. Indeed, proteinases cleave mucosal barrier proteins such as albumin and collagen. Also, they can efficiently hydrolyse host defence proteins, including immunoglobulins, complement proteins, etc. [67,68]. Of the twenty-seven *Candida* isolates tested, only four (14.8%) showed low phospholipase activity and eight (29.6%) showed moderate aspartyl-proteinase activity. These low rates of isolates with enzymatic activity could be associated with the fact that *Candida* is a commensal microorganism of the GI tract. Moreover, most proteases are produced at a more acidic pH than those found in the human intestine.

Comparing the expression of virulence factors between *Candida* species isolated from the group with ASD and the CC group, we could not find significant differences. This fact could be affected in part by the small number of *Candida* isolates yielded from both groups of children. Although the *Candida* species investigated in this study are commensal gut colonizers, they can form biofilm, secrete extracellular enzymes and be hydrophobic. The expression of these virulence factors could be related to a carbohydrate-rich diet. It is hypothesized that glucose enhances the yeast-to-hyphae transition, a fundamental step that leads to increased virulence in *Candida*, by increasing the capacity to proliferate, penetrate the tissues, produce biofilm and escape the immune response of the host [69].

This study has potential limitations that should be taken into account for future work. The balance of the sex distribution is one of them, since in this study the ASD group had a higher proportion of males (78.6%) compared to the control group (58.7%). This disparity is consistent with established epidemiological findings regarding the prevalence of ASD. In addition, other comorbidities and factors such as diet should be considered in the design, as well as increasing the amount of microbiological sampling.

## 5. Conclusions

In summary, we can conclude that there is not a significant correlation between *Candida* colonization in the GI tract of Tunisian children and ASD. *Candida* presence in the gut was not correlated with GI problems in ASD and did not affect ASD severity. However, Tunisian children with ASD presented more GI disorders, particularly constipation, than those without ASD—disturbances that are strongly associated with ASD severity. Further detailed and larger studies are needed to confirm the role of intestinal *Candida* overgrowth and its potential association with the pathogenesis of autism, and examining the whole gut mycobiota in ASD by next-generation sequencing could reveal other associations by identifying fungal species closely related to ASD that are difficult to culture.

## Figures and Tables

**Table 1:** Demographic characteristics and clinical manifestations of Tunisian children from the region of Monastir with ASD (*n* = 28) and CC group (*n* = 46).

Characteristics	ASD Group *n* (%)	CC Group *n* (%)	*p*-Value
Gender			0.08
Male	22 (78.6%)	27 (58.7%)	
Female	6 (21.4%)	19 (41.3%)	
Age (mean ± SD)	7.93 ± 2.09	7.39 ± 2.02	0.119
4–7 years	10 (35.7%)	25 (54.3%)	
8–10 years	18 (64.3%)	21 (45.7%)	
GI disorders	18 (64.3%)	21 (45.7%)	0.119
Constipation	17 (60.7%)	8 (17.4%)	0.000 *
Diarrhoea	1 (3.6%)	1 (2.2%)	1.000
Vomiting	1 (3.6%)	0 (0.0%)	0.378
Oesophageal reflux	1 (3.6%)	0 (0.0%)	0.378
Abdominal pain	7 (25.0%)	14 (30.4%)	0.615
CARS score			
30–36 (mild to moderate ASD)	11 (39.3%)		
37–60 (severe ASD)	17 (60.7%)		

ASD, autism spectrum disorder; CC, control children; CARS, childhood autism rating scale; (*) indicates significant difference between ASD and CC groups.

**Table 2:** In vitro activities of amphotericin B, anidulafungin, micafungin, fluconazole, isavuconazole and ibrexafungerp against *Candida* isolated from the faecal samples of children with autism and control children.

Antifungal Drugs	Species (*n*)	Children with Autism					Control Children				
		MIC (mg/L)		Isolates *n* (%)			MIC (mg/L)		Isolates *n* (%)		
		Range MIC	GM Mean	S	I	R	Range MIC	GM Mean	S	I	R
**Amphotericin B**	*Candida albicans* (17)	0.03–0.125	0.16	7 (41.2)	0	0	0.06–0.5	0.16	10 (58.8)	0	0
	*Candida glabrata* (4)	0.125–0.25	0.18	2 (50)	0	0	0.125–0.25	0.18	2 (50)	0	0
	*Candida parapsilosis* (2)	0.125–4	2.06	1 (50)	0	1 (50)					
	*Candida dubliniensis* (2)	0.03	0.03	1 (50)	0	0	0.03	0.03	1 (50)	0	0
	*Candida guilliermondii* (1)	0.125	0.12	ND	ND	ND					
	*Candida krusei* (1)						0.25	0.25	1 (100)	0	0
**Anidulafungin**	*Candida albicans* (17)	0.008–4	0.58	6 (35.3)	0	1 (5.8)	0.008–0.03	0.02	10 (58.8)	0	0
	*Candida glabrata* (4)	0.06	0.06	2 (50)	0	0	0.03–0.06	0.05	2 (50)	0	0
	*Candida parapsilosis* (2)	0.5–4	2.25	2 (100)	0	0					
	*Candida dubliniensis* (2)	0.03	0.03	ND	ND	ND	0.03	0.03	ND	ND	ND
	*Candida guilliermondii* (1)	0.06	0.06	ND	ND	ND					
	*Candida krusei* (1)						0.03	0.03	1 (100)	0	0
**Micafungin**	*Candida albicans* (17)	0.03–4	0.59	5 (29.4)	0	2 (11.8)	0.016–0.03	0.03	10 (58.8)	0	0
	*Candida glabrata* (4)	0.06–0.125	0.09	0	0	2 (50)	0.06–0.125	0.09	0	0	2 (50)
	*Candida parapsilosis* (2)	0.008–1	0.5	2 (100)							
	*Candida dubliniensis* (2)	0.06	0.06	ND	ND	ND	0.06	0.06	ND	ND	ND
	*Candida guilliermondii* (1)	0.06	0.06	ND	ND	ND					
	*Candida krusei* (1)						0.06	0.06	ND	ND	ND
**Isavuconazole**	*Candida albicans* (17)	0.008–0.016	0.01	ND	ND	ND	0.008–0.03	0.02	ND	ND	ND
	*Candida glabrata* (4)	0.25–1	0.63	ND	ND	ND	0.5–1	0.75	ND	ND	ND
	*Candida parapsilosis* (2)	0.016–0.03	0.02	ND	ND	ND					
	*Candida dubliniensis* (2)	0.008	0.008	ND	ND	ND	0.016	0.016	ND	ND	ND
	*Candida guilliermondii* (1)	0.125	0.125	ND	ND	ND					
	*Candida krusei* (1)						1	1	ND	ND	ND
**Fluconazole**	*Candida albicans* (17)	0.125–64	25.19	3 (16.6)	0	4 (23.5)	0.125–0.25	0.162	10 (58.9)	0	0
	*Candida glabrata* (4)	4–8	6	0	2 (50)	0	4–64	34	0	1 (25)	1 (25)
	*Candida parapsilosis* (2)	0.5	0.5	2 (100)	0	0					
	*Candida dubliniensis* (2)	0.125	0.125	1 (50)	0	0	0.25	0.25	1 (50)	0	0
	*Candida guilliermondii* (1)	1	1	ND	ND	ND					
	*Candida krusei* (1)						32	32	ND	ND	ND
**Ibrexafungerp**	*Candida albicans* (17)	0.016–0.03	0.02	ND	ND	ND	0.016–0.03	0.02	ND	ND	ND
	*Candida glabrata* (4)	0.125–0.25	0.19	ND	ND	ND	0.25	0.2	ND	ND	ND
	*Candida parapsilosis* (2)	0.25–0.5	0.38	ND	ND	ND					
	*Candida dubliniensis* (2)	0.06	0.06	ND	ND	ND	0.03	0.03	ND	ND	ND
	*Candida guilliermondii* (1)	1	1	ND	ND	ND					
	*Candida krusei* (1)			ND	ND	ND	0.125	0.125	ND	ND	ND

MIC, minimal inhibitory concentration; GM, geometric mean; S, susceptible; I, intermediate; R, resistant; ND, not determined.

**Table 3:** Biomass and metabolic activity of *Candida* biofilm.

Children Group	*Candida* Species	Biofilm Biomass Category *n* (%)			Biofilm Metabolic Activity Category *n* (%)		
		HBB	MBB	LBB	HMA	MMA	LMA
ASD	*Candida albicans* (*n* = 7)	3 (42.9)	3 (42.9)	1 (14.3)		4 (57.1)	3 (42.9)
	*Candida parapsilosis* (*n* = 2)			2 (100)			2 (100)
	*Candida glabrata* (*n* = 2)			2 (100)			2 (100)
	*Candida dubliniensis* (*n* = 1)		1 (100)			1 (100)	
	*Candida guilliermondii* (*n* = 1)			1 (100)			1 (100)
CC	*Candida albicans* (*n* = 10)	4 (40)	3(30)	3 (30)	1 (10)	3 (30)	6 (60)
	*Candida glabrata* (*n* = 2)			2 (100)			2 (100)
	*Candida dubliniensis* (*n* = 1)			1 (100)			1 (100)
	*Candida krusei* (*n* = 1)			1 (100)			1 (100)

Isolates were classified as high (HBB), moderate (MBB) and low (LBB) biomass biofilm producers, and with high (HMA), moderate (MMA) and low (LMA) metabolic activity; ASD, autism spectrum disorder; CC, control children.

**Table 4:** Phospholipase and aspartyl-proteinase activities exhibited by *Candida*.

	Species (*n*)					
	*Candida albicans* (17) +/− (%)	*Candida glabrata* (4) +/− (%)	*Candida dubliniensis* (2) +/− (%)	*Candida parapsilosis* (2) +/− (%)	*Candida guilliermondii* (1) +/− (%)	*Candida krusei* (1) +/− (%)
**Phospholipase**						
ASD group	2/5 (11.7/29.4)	0/2 (0/50)	0/1 (0/100)	0/2 (0/100)	0/1 (0/100)	0/0
CC group	2/8 (11.7/47.1)	0/2 (0/50)	0/1 (0/100)	0/0	0/0	0/1 (0/100)
**Aspartyl**-**proteinase**						
ASD group	2/5 (11.7/29.4)	0/2 (0/50)	1/1 (50/50)	0/2 (0/100)	0/1 (0/100)	0/0
CC group	4/6 (23.5/3.5)	0/2 (0/50)	1/1 (50/50)	0/0	0/0	0/1 (0/100)

+, weak enzyme producers; −, not enzyme producers; ASD, autism spectrum disorder; CC, control children.

## Data Availability

The original contributions presented in the study are included in the article/Supplementary Materials, further inquiries can be directed to the corresponding author/s. The raw data supporting the conclusions of this article will be made available by the authors on request.

## Supplementary Materials

The following supporting information can be downloaded at https://www.mdpi.com/article/10.3390/jof10110730/s1, Table S1: Distribution of *Candida* species isolated from stools of children with ASD and control; Table S2: Association between *Candida* presence and clinical parameters among the ASD group; Table S3: Biofilm biomass and metabolic activity values of 27 *Candida* isolates measured at 24 h with Crystal violet (CV) and XTT reduction assays; Table S4: Cell surface hydrophobicity percentage values of 27 *Candida* isolates measured at a wavelength of 600 nm; Figure S1: Agarose gel separation of isolates of *C. albicans* and *C. dubliniensis* identified by multiplex PCR.
