# Supplementary material for: Characterization of Yeast Isolated from the Gut Microbiota of Tunisian Children with Autism Spectrum Disorder
Source: J Fungi (Basel). 2024 Oct 22;10(11):730. doi: 10.3390/jof10110730 (PMC11595294; doi:10.3390/jof10110730)
Supplement: Supplementary file 1 [file jof-10-00730-s001.zip › jof-3237525-supplementary.pdf]

Article

Characterization of yeast isolated from the gut microbiota of Tunisian children with autism spectrum disorder

Mariem Chamtouri [1,2], Abderrahmen Merghni [3] Katherine Miranda-Cadena [4], Nabil Sakly [5], Naoufel Gaddour [6], Clara G. de los Reyes-Gavilán [1,7], Maha Mastouri [2], Elena Eraso [4,] * and Guillermo Quindós [4]

[1] Department of Microbiology and Biochemistry of Dairy Products, Instituto de Productos Lácteos de Asturias (IPLA-CSIC), 33300 Villaviciosa, Spain; myriam.chantouri88@gmail.com (M.C.); greyes_gavilan@ipla.csic.es (C.G.d.L.R.-G.)
[2] Laboratory of Transmissible Diseases and Biologically Active Substances LR99ES27, Faculty of Pharmacy, University of Monastir, Monastir 5000, Tunisia; mastourimaha@yahoo.fr
[3] Laboratory of Antimicrobial Resistance LR99ES09, Faculty of Medicine of Tunis, University of Tunis El Manar, Tunis 1068, Tunisia; abderrahmen_merghni@yahoo.fr
[4] Department of Immunology, Microbiology and Parasitology, Faculty of Medicine and Nursing, University of the Basque Country, UPV/EHU, 48080 Bilbao, Spain; katherine.miranda@ehu.eus (K.M.-C.); guillermo.quindos@ehu.eus (G.Q.)
[5] Laboratory of Medical and Molecular Parasitology-Mycology (code LR12ES08), Department of Clinical Biology B, Faculty of Pharmacy, University of Monastir, Monastir 5000, Tunisia; saklynabil@yahoo.fr
[6] Unit of Child Psychiatry, Monastir University Hospital, Monastir 5000, Tunisia; naoufel.gaddour@gmail.com
[7] Diet, Microbiota, and Health Group, Instituto de Investigación Sanitaria del Principado de Asturias (ISPA), 33011 Oviedo, Spain
* Correspondence: elena.eraso@ehu.eus; Tel.: +34-946-01-83-71

Table S1 Distribution of *Candida s*pecies isolated from stools of children with ASD and control

***Candida* species**	**ASD group (n = 28)**		**CC group (n = 46)**	
	n of isolates	%	n of isolates	%
Candida albicans	7	53.8	10	71.4
Non-*Candida albicans* species	6	46.2	4	28.6
Candida glabrata	2	15.4	2	14.3
Candida parapsilosis	2	15.4	_	_
Candida dubliniensis	1	7.7	1	7.1
Candida guilliermondii	1	7.7	_	_
Candida krusei	_	_	1	7.1
Total	13	46.4	14	30.4

ASD: Autism spectrum disorder; CC: Control children

Table S2 Association between *Candida* presence and clinical parameters among the ASD group

Factor	Positive *Candida* culture (n = 13)	Negative *Candida* culture (n = 15)	P-value
Gender			0.372
Male	9 (69.2%)	13 (86.7%)	
Female	4 (30.8%)	2 (13.3%)	
Age			0.254
4-7 years	3 (23.1%)	7 (46.7%)	
8-10 years	10 (76.9%)	8 (58.3%)	
GI disorders			0.114
Absence	6 (46.2%)	12 (80%)	
Presence	7 (53.8%)	3 (20%)	
Constipation			0.142
Absence	6 (46.2%)	11 (73.3%)	
Presence	7 (53.8%)	4 (26.7%)	
Diarrhoea			1
Absence	0 (0%)	1 (6.7%)	
Presence	13 (100%)	14 (93.3%)	
Vomiting			1
Absence	0 (0%)	1 (6.7%)	
Presence	13 (100%)	14 (93.3%)	
Oesophageal reflux			1
Absence	0 (0%)	1 (6.7%)	
Presence	13 (100%)	14 (93.3%)	
Abdominal pain			0.084
Absence	1 (7.7%)	6 (40%)	
Presence	12 (92.3%)	9 (60%)	
ASD severity			0.488
Mild to moderate	6 (46.2%)	5 (33.3%)	
Severe	7 (53.8%)	10 (66.7%)	

ASD, autism spectrum disorder; GI, gastrointestinal.

Table S3 Biofilm biomass and metabolic activity values of 27 *Candida* isolates measured at 24 h with Crystal violet (CV) and XTT reduction assays

Group	Species	Isolate	Biomass of biofilm			Metabolic activity of biofilm		
			OD	SD	Classification	OD	SD	Classification
ASD	*Candida. albicans*	SC 5314	0.702	0.073	HBB	0.662	0.119	HMA
	Candida. albicans	M1	0.237	0.072	LBB	0.100	0.013	LMA
	Candida. albicans	M3	0.669	0.102	HBB	0.259	0.052	LMA
	Candida. albicans	M5	0.855	0.051	HBB	0.359	0.032	MMA
	Candida. albicans	M14	0.858	0.073	HBB	0.416	0.022	MMA
	Candida. albicans	M20	0.477	0.059	MBB	0.141	0.014	LMA
	Candida. albicans	M23	0.406	0.042	MBB	0.336	0.016	MMA
	Candida. albicans	M25	0.476	0.115	MBB	0.470	0.146	MMA
	Candida. parapsilosis	M4	0.323	0.041	MBB	0.071	0.005	LMA
	Candida. parapsilosis	M13	0.0312	0.012	LBB	0.056	0.012	LMA
	Candida. glabrata	M19	0.135	0.021	LBB	0.230	0.017	LMA
	Candida. glabrata	M24	0.058	0.008	LBB	0.177	0.041	LMA
	Candida. guilliermondii	M11	0.010	0.002	LBB	0.110	0.022	LMA
	Candida. dubliniensis	M26	0.342	0.028	MBB	0.315	0.016	MMA
CC	*Candida. albicans*	M2	0.204	0.070	LBB	0.198	0.031	LMA
	Candida. albicans	M7	0.497	0.023	MBB	0.139	0.016	LMA
	Candida. albicans	M9	0.121	0.015	LBB	0.084	0.020	LMA
	Candida. albicans	M10	0.160	0.051	LBB	0.156	0.049	LMA
	Candida. albicans	M12	0.911	0.051	HBB	0.288	0.029	LMA
	Candida. albicans	M15	0.534	0.045	HBB	0.324	0.020	MMA
	Candida. albicans	M16	0.716	0.099	HBB	0.648	0.103	HMA
	Candida. albicans	M22	0.433	0.063	MBB	0.494	0.034	MMA
	Candida. albicans	M27	0.401	0.053	MBB	0.482	0.013	MMA
	Candida. albicans	M30	0.660	0.072	HBB	0.223	0.022	LMA
	Candida. glabrata	M6	0.145	0.051	LBB	0.184	0.021	LMA
	Candida. glabrata	M28	0.176	0.039	LBB	0.244	0.009	LMA
	Candida. krusei	M18	0.257	0.037	LBB	0.148	0.009	LMA
	Candida. dubliniensis	M21	0.231	0.061	LBB	0.173	0.004	LMA

The isolates of *Candida* were classified as high biomass (HBB), moderate biomass (MBB) or low biomass (LBB) biofilm producers, and with high metabolically activity (HMA), moderate metabolic activity (MMA) or low metabolically activity (LMA) biofilm producer. OD: Optical density; SD: Standard deviation; ASD: Autism spectrum disorder; CC: Control children.

Table S4 Cell surface hydrophobicity percentage values of 27 *Candida* isolates measured at a wavelength of 600 nm

Group	Species	Isolate	Cell surface hydrophobicity (%)	
			Mean ± SD	Category
ASD	*Candida albicans*	M1	26.86 ± 4.84	Moderately hydrophobic
	Candida albicans	M3	24.57 ± 6.39	Moderately hydrophobic
	Candida albicans	M5	22.52 ± 7.84	Moderately hydrophobic
	Candida albicans	M14	22.27 ± 7.99	Moderately hydrophobic
	Candida albicans	M20	19.87 ± 5.59	Hydrophilic
	Candida albicans	M23	20.69 ± 6.91	Moderately hydrophobic
	Candida albicans	M25	18.25 ± 6.84	Hydrophilic
	Candida parapsilosis	M4	19.54 ± 6.98	Hydrophilic
	Candida parapsilosis	M13	21.84 ± 6.76	Moderately hydrophobic
	Candida glabrata	M19	21.16 ± 7.45	Moderately hydrophobic
	Candida glabrata	M24	13.35 ± 21.25	Hydrophilic
	Candida guilliermondii	M11	7.28 ± 2.48	Hydrophilic
	Candida dubliniensis	M26	32.5 ± 9.16	Moderately hydrophobic
CC	*Candida albicans*	M2	6.67 ± 2.76	Hydrophilic
	Candida albicans	M7	7.98 ± 0.68	Hydrophilic
	Candida albicans	M9	11.19 ± 5.46	Hydrophilic
	Candida albicans	M10	7.66 ± 1.43	Hydrophilic
	Candida albicans	M12	7.74 ± 1.68	Hydrophilic
	Candida albicans	M15	5.92 ± 1.26	Hydrophilic
	Candida albicans	M16	7.69 ± 1.08	Hydrophilic
	Candida albicans	M22	9.20 ± 2.61	Hydrophilic
	Candida albicans	M27	7.41 ± 2.01	Hydrophilic
	Candida albicans	M30	12.77 ± 1.33	Hydrophilic
	Candida glabrata	M6	4.70 ± 2.80	Hydrophilic
	Candida glabrata	M28	8.53 ± 3.19	Hydrophilic
	Candida krusei	M18	7.15 ± 1.98	Hydrophilic
	Candida dubliniensis	M21	27.39 ± 6.85	Moderately hydrophobic

The isolates of *Candida* were classified as moderately hydrophobic and hydrophilic. SD: Standard deviation; ASD: Autism spectrum disorder; CC: Control children.

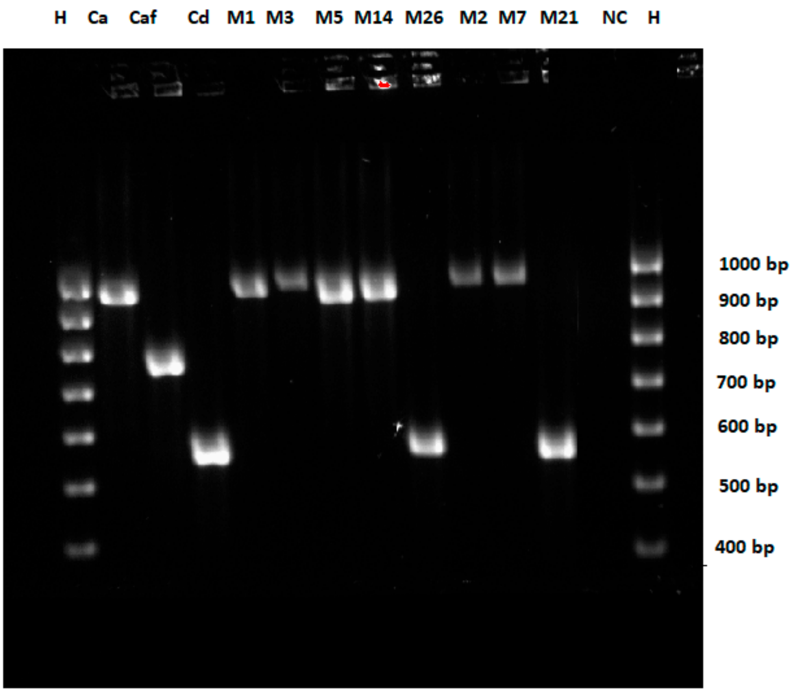

Figure S1 Agarose gel separation of isolates of *C. albicans* and *C. dubliniensis* identified by multiplex PCR. Ca, *C. albicans* control with expected DNA fragments of 941 pb; Caf, *C. africana* control with expected DNA fragments of 700 pb; Cd, *C. dubliniensis* control with expected DNA fragments of 569 pb; M1, M3, M5, M14, M2 and M7 isolates identified as *C. albicans*; M21 and M26, isolates identified as *C. dubliniensis*; H, marker HyperLadder 100 bp; NC, negative control.
